# Prediction of Vertical Ground Reaction Forces Under Different Running Speeds: Integration of Wearable IMU with CNN-xLSTM

**DOI:** 10.3390/s25041249

**Published:** 2025-02-18

**Authors:** Tianxiao Chen, Datao Xu, Zhifeng Zhou, Huiyu Zhou, Shirui Shao, Yaodong Gu

**Affiliations:** 1 Faculty of Sports Science, Ningbo University, Ningbo 315211, China; 2 Faculty of Engineering, University of Pannonia, 8200 Veszprem, Hungary; 3 Faculty of Engineering, University of Szeged, 6720 Szeged, Hungary

**Keywords:** running, ground reaction force, wearable IMU, deep learning, biomechanics prediction, xLSTM

## Abstract

Traditional methods for collecting ground reaction forces (GRFs) mainly use lab force plates. Previous research broke this pattern by predicting GRFs with deep learning and data from IMUs like joint acceleration. Joint angle, as a geometric, is easier to collect than acceleration outdoors with cameras. LSTM is one of the deep learning models that have shown good performance in biomechanical studies. xLSTM, as an optimized version of LSTM, has not been used in biomechanical studies and no research has predicted GRFs during running solely using lower limb joint angles. This study collected lower-limb joint angle and vertical ground reaction force data at five speeds from 12 healthy male runners with Xsens sensors. Datasets including three joints and three planes were set as the inputs of four deep learning models for vertical-GRF prediction. CNN-xLSTM consistently performed best in the four deep learning models when different datasets were input (R^2^ = 0.909 ± 0.064, MAPE = 2.18 ± 0.09, rMSE = 0.061 ± 0.008), and the performance was at a relatively high level at the five speeds. The current findings may contribute to a new GRF measurement and provide a reference for future real-time motion detection and sport injury prediction.

## 1. Introduction

Running, as a widely popular form of exercise, has been deeply favored by the masses due to its simplicity, ease of implementation, and significant effects. A substantial amount of research in sport training and biomechanics has been conducted around this fundamental movement form [1,2]. In biomechanics, the gait phase of running is generally divided into the stance phase and the swing phase [3,4]. The ground reaction force (GRF), as a crucial factor that drives runners moving forward, has attracted extensive attention from biomechanics researchers. Accurate measurement of the ground reaction force during running is of great significance. Biomechanical analysis, through measuring the vertical ground reaction force during running, helps to intuitively understand the force distribution during running, thereby optimizing running technique, such as adjusting the running posture and foot strike pattern, reducing the impact force, and improving running efficiency [5]. At the same time, this measurement also aids in preventing sport injuries, assessing injury risks, and formulating personalized preventive measures. In addition, the vertical ground reaction force is a key parameter in scientific research, providing data support for the development of sport science, and is of great importance for the training guidance of professional athletes and coaches. Ultimately, by optimizing technique, running efficiency can be improved, enhancing the runner’s sporting experience and stimulating their enthusiasm for continued participation [6,7]. Compared with GRFs in other directions, the magnitude of the vertical ground reaction force (vertical GRF) directly influences the propulsive efficiency and energy conversion of running [8]. Previous studies have shown that, under the rear-foot strike running pattern, the vertical-GRF curve typically exhibits a double-peak trend [9,10,11].

Traditionally, the measurement of GRFs has primarily relied on specialized force plates in laboratories. Although the data from force plates is highly valuable for in-depth analysis of a runner’s running posture, assessment of running efficiency, and prediction of sport injury risks, the high equipment costs, spatial limitations, and lack of flexibility in data collection have become limitations in their widespread application [12]. Therefore, exploring a method that can measure GRFs in non-laboratory environments through convenient and low-cost means is of great significance for advancing sport science research and sport training practice [13,14,15]. Such a method can not only reduce the cost and improve the flexibility of data collection but also enable runners and coaches to conduct real-time monitoring and analysis in daily training or home environments, thereby better guiding training and preventing sport injuries [16,17,18].

With the development of wearable inertial measurement units (IMUs) and deep learning, more and more biomechanical studies have used wearable IMUs to collect kinematics and dynamics and then input these data into machine learning and DL models for the classification, recognition, and prediction of athletic performance and sport injuries [19,20,21,22]. Previous studies have directly or indirectly predicted ground reaction forces in running using machine learning and deep learning algorithms, which contributed to breaking the traditional mode of measuring GRFs with force plates in laboratories [23,24,25,26].

Long Short-Term Memory (LSTM), as a special recurrent neural network structure, has been applied to analysis and prediction tasks in the field of biomechanics in previous research. The gating mechanism of LSTM is capable of capturing complex long-term dependencies, which is particularly important when predicting the relationship between the ground reaction force and joint angle changes during the running stance phase. LSTM can handle long-sequence data, avoiding gradient vanishing or exploding problems, making it suitable for processing longer sequences of data. Moreover, LSTM has a good generalization ability and can be combined with other network structures, such as CNN, to improve prediction accuracy and robustness, making it applicable to different runners and environments, thereby broadening the application scope of GRF prediction [27,28]. Alcantara et al. [29] and Donahue et al. [30] predicted GRFs accurately with an LSTM-based model. The Extended LSTM (xLSTM) network was proposed by M. Beck and his team, the founders of LSTM [31]. As an extension of LSTM, it encompasses two variants: scalar LSTM (sLSTM) and matrix memory LSTM (mLSTM). The sLSTM block retains LSTM’s sequential processing and optimizes gating through fine-grained control, making it suitable for subtle temporal variations. The mLSTM block processes all the token sequences simultaneously, enhancing memory and parallel processing by extending LSTM’s vector operations to matrix operations. The two different modules can be flexibly combined within the xLSTM architecture to balance parallelism and sequential modeling [31,32]. Though xLSTM has been used in previous studies for the prediction of time-series data and has demonstrated good performance, there are currently no studies in the field of biomechanics that use xLSTM to analyze kinematics and kinetics [33,34,35].

Previous studies have mainly used kinematic data, such as acceleration, from IMUs as inputs of deep learning models. However, although these studies have accurately estimated the ground reaction force during running, there have been no studies that have used lower-limb joint angles as a single input to predict the GRF. With the advancement of machine learning and deep learning algorithms, complex models are often used for visual data analysis and real-time recognition [36]. Joint angle is not only a type of geometric data that can be captured in real time through imaging equipment and algorithms but is also a basic type of kinematic data in sport biomechanics that has attracted much research attention [37,38]. Based on the above introduction, the aim of this study was to develop an xLSTM-based deep learning model to predict the vertical ground reaction force during the stance phase of running by inputting the angle data of lower-limb joints (ankle, hip, and knee) on three planes (sagittal, frontal, and transversal) and explore the influence of the angles of different joints and different motion planes on the accuracy of prediction results. This study may provide alternatives to break the traditional pattern of collecting ground reaction forces by using force plates in the laboratory. We assumed that the prediction would work best when all three joint angles on all three planes were input. The main contributions of this study are as follows.

1. We develop a deep learning model that can accurately predict the vertical ground reaction force during the stance phase of running by inputting the joint angles of the lower limbs.

2. We explore the impact of different joint angles on different planes on the prediction results of vertical ground reaction forces.

3. We test the predictive performance of the developed model at five different running speeds.

## 2. Procedure

**Figure 1:**
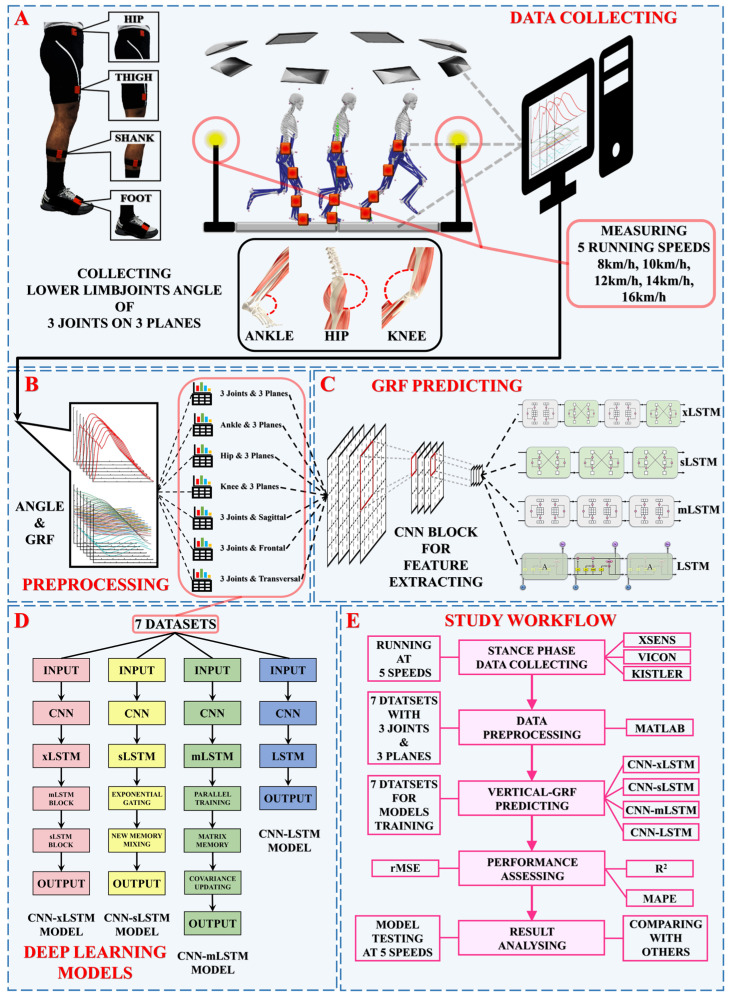
An illustration of the study’s structure. The study was divided into 3 parts. (**A**) The data collection procedure. Xsens sensors and a Vicon 3D motion capture system were used to collect the joint angle and vertical ground reaction force data. (**B**) The data preprocessing procedure. (**C**) The GRF prediction procedure. The datasets were set as the input of 4 deep learning models (CNN-xLSTM, CNN-sLSTM, CNN-mLSTM, and CNN-LSTM) in order to compare and analyze the performance of models in the vertical-GRF prediction tasks. (**D**) The process for each deep learning model. (**E**) The workflow of the entire study.

The study was divided into 3 main parts. First, lower-limb joint angle and ground reaction force data were collected from 12 healthy male runners through a Vicon three-dimensional motion capture system, Kistler force plates, and Xsens sensors during the running stance phase. Second, the collected data were preprocessed and categorized using different joints and different planes. Third, angles from different joints and planes were used to train 4 deep learning models (CNN-xLSTM, CNN-sLSTM, CNN-mLSTM, and CNN-LSTM) to predict the vertical ground reaction forces. The workflow of the study is shown in Figure 1.

### 2.1. Data Collection and Preprocessing

The vertical-GRF data and the lower-limb joint angle data were collected in the Biomechanics Laboratory of Ningbo University. Twelve healthy male runners (age: 22.5 ± 0.86 years; body mass: 72.5 ± 9.55 kg; height: 1.78 ± 0.77 m) were recruited to participate in the study. The subject screening criteria were as follows: (1) participants must have no history of serious lower-limb surgery or any other injury variables in the past six months that would interfere with the study; and (2) there must be no other factors that would affect athletic performance. All participants were informed of the purpose, requirements, and procedures of the experiment and signed a written informed consent form. This study complied with the principles laid down in the Declaration of Helsinki. Ningbo University’s Ethics Committee accepted the study protocol (Approval Number: TY2024037), and all subjects supplied and signed a written informed permission form.

Vertical GRFs during running were collected in this study through a Vicon three-dimensional motion capture system (Vicon Metrics Ltd., version 2.14.0, Oxford, UK) and Kistler force plates. The sampling frequency was set to 200 Hz and 1000 Hz, respectively. Two photoelectric gates were placed on both sides of the Kistler force plates, and the time it took runners to pass the force plate was recorded and converted to the running speed (8 km/h, 10 km/h, 12 km/h, 14 km/h, and 16 km/h) [39]. Each runner was required to run through the Vicon–Kistler–photoelectric gate system with Xsens sensors 10 times at each speed. All runners were required to wear Xsens motion capture sensors and to wear designated clothes and running shoes while running to collect the angle of 3 joints (hip, knee, and ankle) on 3 planes (sagittal, frontal, and transversal). The Xsens sensors (Xsens, Henderson, NV, USA) were set on the hip, thigh, shank, and foot of each runner (Figure 1). All runners were required to perform the running task with the rear-foot strike pattern and were allowed adequate rest after each running task.

The stance phase of running was defined as the period from the initial contact of the right heel with the ground (when the GRF collected by the force platform exceeded 10 N) to the complete liftoff of the right forefoot from the ground [40]. The phase was divided into a period of 0–100% in this study. A fourth-order Butterworth low-pass filter was used to process the collected ground reaction force and joint angle data, with cutoff frequencies set at 10 Hz and 20 Hz, respectively. The filtered data were imported into MATLAB (Visual R2022a, MathWorks, Natick, MA, USA), and we used a MATLAB script to perform an interpolation calculation, expanding the data to 101 points corresponding to 0–100% of the running stance phase. Data on missing instances and eliminated outliers were checked through MATLAB scripts to ensure the accuracy of the dataset. After the preprocessing procedure, 530 sets of one-to-one corresponding vertical-GRF and joint angle data were put into the final dataset. To investigate the impact of different input data on the prediction results, the calculated joint angles were classified as follows and used as different inputs:

1. M_1 (3*Joints*, 3*Planes*)_ = 530 × 909 _(3*joints* × 3*planes* × 101*angles*)_;
2. M_2 (*Ankle*, 3*Planes*)_ = 530 × 303 _(1*ankle joint* × 3*planes* × 101*angles*)_;
3. M_3 (*Hip*, 3*Planes*)_ = 530 × 303 _(1*hip joint* × 3*planes* × 101*angles*)_;
4. M_4 (*Knee*, 3*Planes*)_ = 530 × 303 _(1*knee joint* × 3*planes* × 101*angles*)_;
5. M_5 (3Joints, Sagittal)_ = 530 × 303 _(3joints × 1sagittal plane × 101angles)_;
6. M_6 (3Joints, Frontal)_ = 530 × 303 _(3joints × 1frontal plane × 101angles)_;
7. M_7 (3Joints, Transversal)_ = 530 × 303 _(3joints × 1transversal plane × 101angles)_.

### 2.2. Deep Learning Models

**Figure 2:**
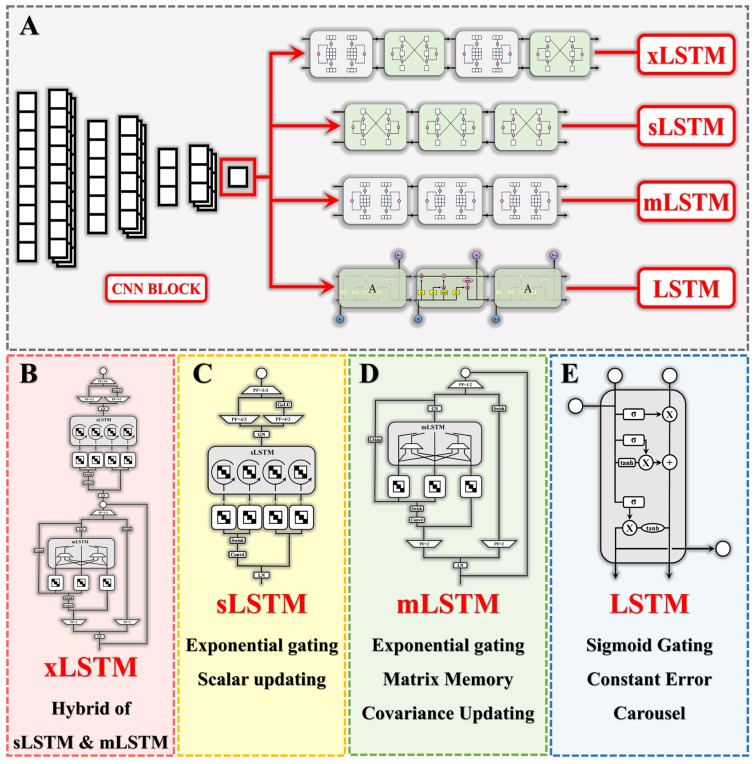
The structure of the 4 deep learning models. (**A**) The CNN block was combined with xLSTM, sLSTM, mLSTM, and LSTM for feature extraction and vertical-GRF prediction. (**B**) The basic unit of xLSTM, which is a hybrid of sLSTM and mLSTM. (**C**) The basic unit of sLSTM. (**D**) The basic unit of mLSTM. (**E**) The basic unit of LSTM.

A CNN-xLSTM network, a CNN-sLSTM network, a CNN-mLSTM network, and a CNN-LSTM network were developed in this study for vertical-GRF prediction. The development, training, and validation of the 4 deep learning models were conducted in PyCharm (V2024.2.3, JetBrains, Prague, Czech Republic). The structure of the deep learning models is shown in Figure 2.

#### 2.2.1. Convolutional Neural Networks (CNNs)

The basic structure of a Convolutional Neural Network (CNN) comprises an input layer, convolutional layers, pooling layers, fully connected layers, and an output layer. The input layer receives the original data, the convolutional layers extract features using multiple convolution kernels to generate feature maps, the pooling layers reduce the dimensionality of the feature, decreasing the computational load and preventing overfitting, the fully connected layers transform the output of the pooling layers into a probability distribution for classification results, and the output layer produces the final classification labels [41,42].

A CNN block with a convolutional kernel size of 3 and a pooled layer size of 2 was set and combined with 4 deep learning model blocks (xLSTM, sLSTM, mLSTM, and LSTM) in this study to extract temporal features of the joint angle and vertical ground reaction forces during the stance phase. The output of the CNN was set as the input for subsequent models in this study.

#### 2.2.2. Long Short-Term Memory (LSTM)

The LSTM model achieves the effective capture and memorization of long-sequence messages by introducing a cell status and three logic gates (a forget gate, an input gate, and an output gate) that control message transmission [28]. The main characteristic of this model lies in its unique “gating mechanism”, with the primary algorithmic formula as follows:

C_t_ = f_t_ ∙ C_t−1_ + i_t_ ∙ z_t_ (1)

where C_t_ represents the cell state at time t; f_t_ represents the output of the forget gate at time t; C_t−1_ represents the cell state at the previous time step; i_t_ represents the output of the input gate at time t; and z_t_ represents the candidate cell state at time t.

The nn LSTM class from the PyTorch deep learning framework was utilized to implement this LSTM layer structure. This class provides all the necessary functionalities for constructing layers, including parameter initialization and forward propagation.

#### 2.2.3. Extended Long Short-Term Memory (xLSTM)

xLSTM is actually a hybrid model of two variants: scalar LSTM (sLSTM) and matrix LSTM (mLSTM). sLSTM retains the memory mixing function of traditional LSTM and supports state tracking, making it suitable for tasks that require the capture of subtle changes in time-series data, while mLSTM introduces a normalizer state to track the product of the input gate and the future forget gate. Additionally, mLSTM achieves full parallelism, enabling the efficient processing of large-scale data and making it suitable for tasks requiring a fast response and high-performance computing [31]. The two blocks can be switched and selected by modifying the ‘s’ or ‘m’ module in the model definition code in Pycharm.

sLSTM improves upon the standard LSTM algorithm through its unique exponential gating mechanism. This variant introduces an exponential function as the activation function of the model to control information flow, making the activation of the input gate and forget gate more efficient and stable. Based on this mechanism, the forward propagation algorithm of sLSTM is as follows:

n_t_ = f_t_ ∙ n_t−1_ + i_t_ (2)

where n_t_ represents a normalization state, which sums the product of the input gate and all future forget gates; f_t_ represents the activation value of the forget gate, which is used to regulate the amount of information inherited from the previous time step t − 1; n_t−1_ represents the state of the previous time step; and i_t_ represents the new information added at the current time step.

(3) $${h_{t}=o_{t}\cdot \;\tilde{h}}_{t},$$

(4) $${\tilde{h}}_{t}=c_{t}/n_{t}$$

where $h_{t}$ represents the output state at a time step; $o_{t}$ represents the activation value of the output gate, which is used to regulate the amount of output from the cell; ${\tilde{h}}_{t}$ represents the candidate output state used to adjust the activation level; and $c_{t}$ represents the internal state of the cell or the “memory cell” state, which is typically used to store long-term information.

(5) $$i_{t}=\exp\left({\tilde{i}}_{t}\right),$$

(6) $$f_{t}=\exp\left({\tilde{f}}_{t}\right)$$

where $i_{t}$ and $f_{t}$ represent the activation values of the input gate and forget gate, respectively, after being transformed by the exponential function exp, and ${\tilde{i}}_{t}$ and ${\tilde{f}}_{t}$ represent the activation values before the transformation. This process ensures that the output values of the input gate and forget gate are positive and uses these outputs for subsequent nonlinear activation and the regulation of information flow [31].

mLSTM extends the vector operations in the original LSTM algorithm to matrix operations, significantly enhancing the model’s memory capacity and parallel processing capability. The algorithm for updating the memory cell through matrix operations in mLSTM is as follows:

(7) $$C_{t}=f_{t}⊙C_{t-1}+i_{t}⊙\;\tanh\;(W_{c}\cdot \;[h_{t-1},\;x_{t}]+b_{c})$$

where $C_{t}$ represents the memory cell matrix at the current time step, ⊙ denotes element-wise multiplication, $i_{t}$ represents the input gate matrix, $\left[h_{t-1},\;x_{t}\right]$ is the matrix formed by concatenating the hidden state $h_{t-1}$ from the previous time step and the input $x_{t}$ at the current time step, and $b_{c}$ is a bias term. The function of the hidden state in mLSTM is:

(8) $${\tilde{h}}_{t}=C_{t}\;q_{t}/\max\left\{\left|n_{t}^{⊤}q_{t}\right|,1\right\}$$

where $C_{t}$ represents the memory cell matrix at the current time step, ${\tilde{h}}_{t}$ represents the hidden state at the current time step, and $q_{t}$ denotes the query input. The parallelization capability of mLSTM significantly improves the computational efficiency when processing long sequences by eliminating memory mixing, enabling the parallel capture and processing of high-dimensional information in tokens and, thus, accelerating the training and inference processes [31].

### 2.3. Model Training and Validation

#### 2.3.1. Model Training

The 7 datasets mentioned in Section 2.1 were used as the input of 4 deep learning models. The Min–Max normalization technique was employed to normalize the data, and the algorithm formula for this process is as follows:

(9) $$x^{\prime }=\frac{x-\min}{\max-\min}$$

where $x^{\prime }$ represents the value of a single data point, min is the minimum value in the column of data, and max is the maximum value in the column of data. This technique scales the original data to a range between 0 and 1. This method not only preserves the original distribution of the data but also unifies the scale of the data, making different features or variables comparable.

#### 2.3.2. Model Validation

K-fold cross-validation was chosen in this study for model validation and to overcome the overfitting problem. Through 10-fold cross-validation (K = 10), the shuffled dataset was evenly divided into 10 subsets of equal size, and 10 iterations were performed. In each iteration, 9 subsets were selected as the training set, and the remaining subset was used as the test set. The squared correlation coefficient (R^2^), the Mean Absolute Percentage Error (MAPE), and the root Mean Squared Error (rMSE) between the predicted values and the actual values were calculated by the matplotlib library with Python code to evaluate the performance of the 4 models in the task of vertical-GRF prediction. The formula for R^2^ is as follows:

(10) $$R^{2}=1-\frac{\sum ({\hat{y}}^{\left(i\right)}-y^{\left(i\right)}{)}^{2}}{\sum_{i}\;(\overset{-}{y}-y^{\left(i\right)}{)}^{2}}$$

where $y^{\left(i\right)}$ represents the true value, ${\hat{y}}^{\left(i\right)}$ represents the predicted value, $\overset{-}{y}$ represents the sample mean, $\sum ({\hat{y}}^{\left(i\right)}-y^{\left(i\right)}{)}^{2}$ represents the error generated by the predictions, and $\sum_{i}\;(\overset{-}{y}\;-y^{\left(i\right)}{)}^{2}$ represents the error generated by the mean. The R^2^ is better when it is larger. When the prediction model makes no errors, the R^2^ reaches its maximum value of 1.

The formula for MAPE is as follows:

(11) $$\mathrm{MAPE}=\frac{100\%}{n}\sum_{i=1}^{n}\;|\frac{{\hat{y}}_{i}-y_{i}}{y_{i}}|$$

where $y_{i}$ is the true value and ${\hat{y}}_{i}$ represents the predicted value. The range is [0, +∞). An MAPE of 0% indicates a perfect model, while an MAPE greater than 100% indicates a poor model.

The formula for the rMSE is as follows:

(12) $$\mathrm{rMSE}=\sqrt{\frac{1}{n}\sum_{i=1}^{n}\;\;(y_{i\;}-{\hat{y}}_{i}{)}^{2}}$$

where n represents the number of samples, $y_{i}$ represents the actual value of the data, and ${\hat{y}}_{i}$ represents the predicted value of the data. A smaller MSE value indicates a smaller difference between the predicted values and the actual values, implying more accurate predictions.

## 3. Results

### 3.1. Parameters of Deep Learning Models

**Figure 3:**
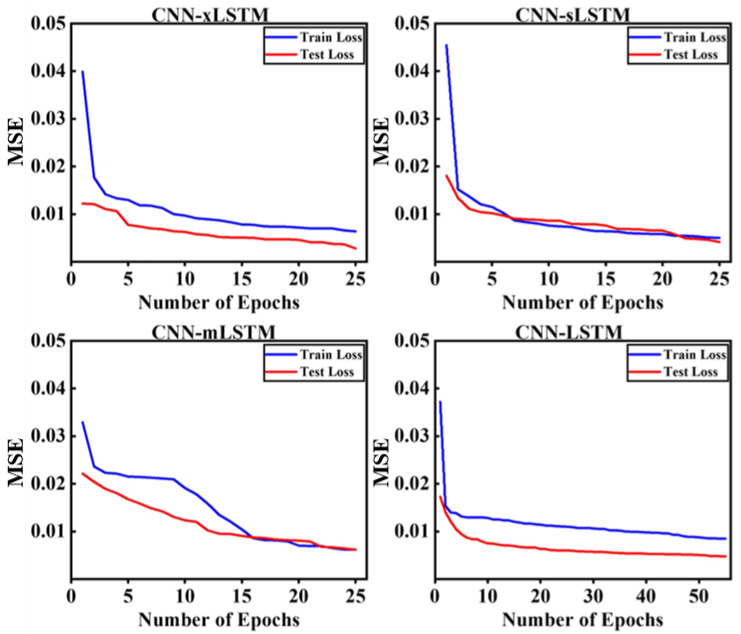
The visualization of the training loss and testing loss curve for each deep learning model in each epoch.

The seven datasets mentioned in the data processing section were used as the input of four deep learning models (CNN-xLSTM, CNN-sLSTM, CNN-mLSTM, and CNN-LSTM). After training and testing multiple combinations of variant structures, a CNN-xLSTM model that integrates an mLSTM module with an sLSTM module was ultimately constructed in this study. Table 1 shows the optimal parameter configuration of each model. The training loss and testing loss with the configuration are shown in Figure 3.

### 3.2. Prediction Results and Model Performance

**Figure 4:**
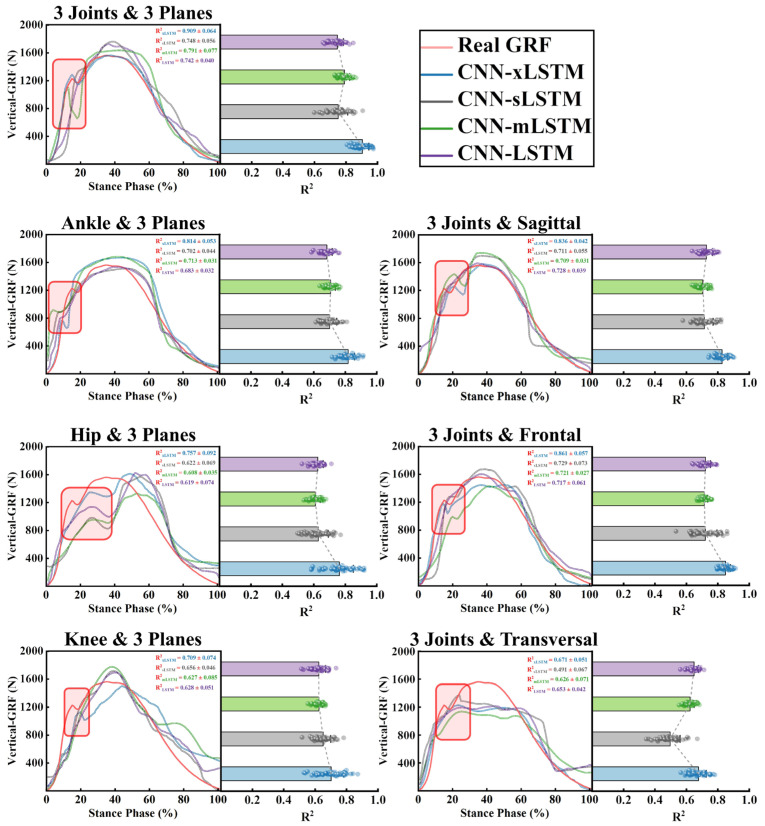
Visualization of the vertical-GRF prediction results obtained from different inputs (M_1 (3*Joints*, 3*Planes*)_, M_2 (*Ankle*, 3*Planes*)_, M_3 (*Hip*, 3*Planes*)_, M_4 (*Knee*, 3*Planes*)_, M_5 (3*Joints*, *Sagittal*)_, M_6 (3*Joints*, *Frontal*),_ and M_7 (3*Joints*, *Transversal*)_). Each diagram shows the prediction results and the R^2^ of the 4 different deep learning models.

The vertical-GRF prediction results of training four deep learning models with data from different joints and planes (M_1 (3*Joints*, 3*Planes*)_, M_2 (*Ankle*, 3*Planes*)_, M_3 (*Hip*, 3*Planes*)_, M_4 (*Knee*, 3*Planes*)_, M_5 (3*Joints*, *Sagittal*)_, M_6 (3*Joints*, *Frontal*),_ and M_7 (3*Joints*, *Transversal*)_) are shown in the Figure 4. Table 2 shows the R^2^, MAPE, and rMSE of the four models in each prediction task. In Figure 4 and Table 2, when the dataset M_1 (3*Joints*, 3*Planes*)_ was used as the input dataset, the fitting effect of the prediction results of the four models was the best (R^2^_xLSTM_ = 0.909 ± 0.064, R^2^_sLSTM_ = 0.748 ± 0.056, R^2^_mLSTM_ = 0.791 ± 0.077, and R^2^_LSTM_ = 0.742 ± 0.040), which means that the angles of the ankle, hip, and knee joints on all three planes during the running stance phase made the biggest contribution to accurate vertical-GRF prediction results.

**Figure 5:**
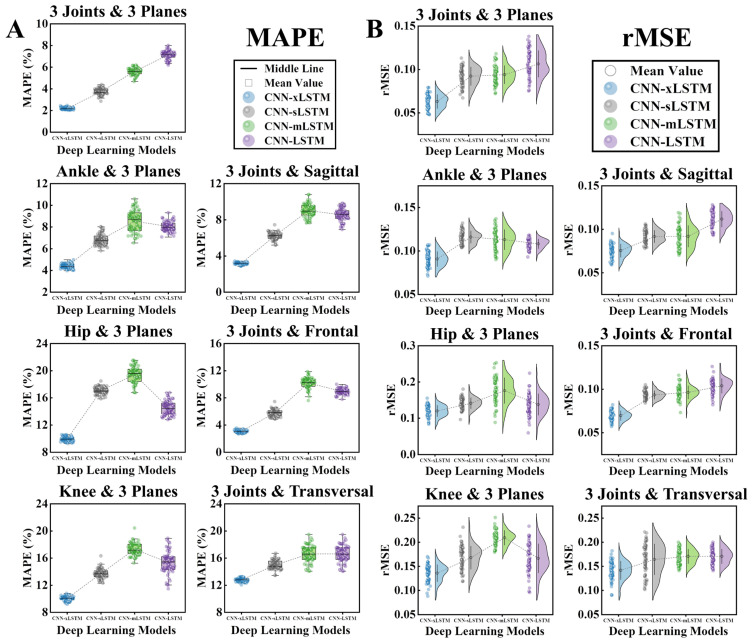
Visualization of the MAPE and rMSE obtained from 4 deep learning models (CNN-xLSTM, CNN-sLSTM, CNN-mLSTM, and CNN-LSTM). The lower the MAPE and rMSE, the better the performance of models in fitting the vertical-GRF curve. (**A**) MAPE of each model with different inputs. (**B**) rMSE of each model with different inputs.

In addition, when the input datasets were M_2 (*Ankle*, 3*Planes*)_, M_5 (3*Joints*, *Sagittal*)_, and M_6 (3*Joints*, *Frontal*)_, the four models also showed a relatively good performance in fitting the vertical-GRF curve. When the input datasets were M_3 (*Hip*, 3*Planes*)_, M_4 (*Knee*, 3*Planes*)_, and M_7 (3*Joints*, *Transversal*)_, the fitting effect of the four models on the vertical ground reaction force curve was not ideal. In Figure 4, Figure 5, and Table 2, when the seven datasets were input separately, the CNN-xLSTM model consistently showed the best fitting effect for the vertical-GRF curve among the four models, i.e., the highest R^2^ value (R^2^ = 0.909 ± 0.064), the lowest MAPE value (MAPE = 2.18 ± 0.09), and the lowest rMSE value (rMSE = 0.061 ± 0.008).

### 3.3. Result Validation

**Figure 6:**
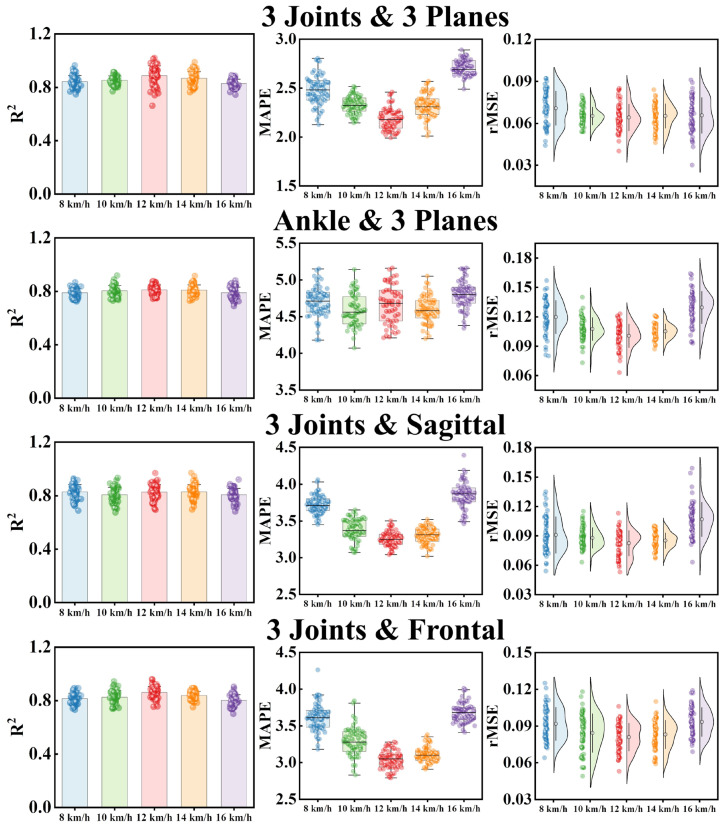
The visualization of the R^2^, MAPE, and rMSE of CNN-xLSTM at 5 running speeds in the 4 cases that fitted the vertical-GRF curve better.

Joint angle and vertical ground reaction force data at five different running speeds (8 km/h, 10 km/h, 12 km/h, 14 km/h, and 16 km/h) were collected in this study. The CNN-xLSTM model with an sLSTM block and an mLSTM block showed the best vertical-GRF prediction performance among the four models (as described in Section 2.2). Datasets at five speeds were input into CNN-xLSTM to validate the accuracy of the predicted vertical-GRF values at different running speeds, and the four cases with better prediction performance (M_1 (3*Joints*, 3*Planes*),_ M_2 (*Ankle*, 3*Planes*)_, M_5 (3*Joints*, *Sagittal*)_, and M_6 (3*Joints*, *Frontal*)_) are discussed in this section. The R^2^, MAPE, and rMSE of CNN-xLSTM at five running speeds are shown in Table 3 and Figure 6. In Table 3, the performance of CNN-xLSTM in the vertical-GRF prediction tasks was at a relatively stable level at all five running speeds. Each case performed best at the speed of 12 km/h, and the case of three joints and three planes made the biggest contribution to the prediction results (R^2^ = 0.879 ± 0.068, MAPE = 2.19 ± 0.12, rMSE = 0.063 ± 0.010). The performance of the CNN-xLSTM model developed in this study is compared with the studies mentioned in this article in Table 4.

## 4. Discussion

The purpose of this study was to develop an xLSTM-based deep learning model to predict the vertical ground reaction force during the stance phase of running by inputting the angles of the lower-limb joints (ankle, hip, and knee) on three planes (sagittal, frontal, and transversal) and to explore the influence of the angles of different joints and different motion planes on the accuracy of prediction results. We first collected lower-limb joint angles and vertical ground reaction forces at five speeds from 12 healthy male runners during the running stance phase with Xsens sensors. The collected data were divided into seven datasets, including three lower-limb joints (ankle, hip, and knee) and three planes (sagittal, frontal, and transversal), which were set as the input datasets for each of the four deep learning models (CNN-xLSTM, CNN-sLSTM, CNN-mLSTM, and CNN-LSTM). The CNN-xLSTM model showed the best performance in the vertical-GRF prediction tasks among the four models (R^2^ = 0.909 ± 0.064, MAPE = 2.18 ± 0.09, rMSE = 0.061 ± 0.008).

### 4.1. Contribution of Different Joint Angles

Most previous studies used joint acceleration or angular velocity from a wearable IMU as the input of machine learning or deep learning models to predict the ground reaction force [25,26,29]. However, there are few studies that predicted ground reaction forces with joint angles alone. The Xsens wearable sensors that were used in this study allowed us to collect joint angle data directly, skipping the process of conversion through other variables. As a type of intuitive geometric data, joint angles can be captured directly by the cameras and identified by a specific algorithm then set as the input of subsequent algorithms, which may help to break away from the limitations of the traditional mode of measuring ground reaction forces with force plates in the laboratory [12]. Lower-limb joint angle data from three joints and three planes were used as the input into deep learning models in this study to predict vertical GRFs during the running stance phase. In the seven inputs mentioned in Section 2.1, when M_1 (3*Joints*, 3*Planes*)_ was input, all models achieved the best prediction performance when the seven datasets were input separately, indicating that the data from the three joints on all three planes contributed the most to accurate vertical ground reaction force prediction results. At the same time, we also found that, among the three joints, the performance of the four models was better than that of the other two joints when the angles of the ankle were entered on the three planes. On the three planes, when the joint angles on the sagittal and frontal planes were input, the prediction performance of the model was also relatively good (Figure 4). These findings not only reveal the influence of different lower-limb joint angles on different planes on the accurate prediction of vertical ground reaction forces, but also provide a reference for the setting of sport analysis equipment and the rehabilitation and training of athletes or sport injury patients [18,43]. The joint angles on the sagittal and frontal planes have a significant impact on predicting the ground reaction force during running. Changes in joint angles on the sagittal plane are directly related to lower-limb propulsion movements and the force output, affecting the direction and magnitude of the ground reaction force, whereas the joint angles on the frontal plane, although small, are crucial for maintaining running stability and also have an indirect influence on the ground reaction force. When data from joint angles on all three planes are considered comprehensively, deep learning models can more fully capture the lower-limb movement status and accurately predict the ground reaction force [44]. At the same time, the joint angles on the sagittal and frontal planes have independent predictive capabilities, reflecting information on lower-limb propulsion and stability, respectively. Understanding these joint angles is of great significance for optimizing running posture and improving running efficiency.

### 4.2. Performance of CNN-xLSTM

As an optimized model of LSTM, xLSTM was proposed by Sepp Hochreiter and his team. xLSTM combines the advantages of the sLSTM and mLSTM variants in dealing with different tasks and can freely select and combine modules to cope with different tasks through the Python language [31,32]. Since its proposal, xLSTM has demonstrated powerful performance in prediction tasks in many fields [34,45,46]. In this study, we developed a CNN-xLSTM model to predict vertical GRFs during running. CNN-LSTM was constructed by connecting a CNN module to an xLSTM containing an sLSTM module and an mLSTM module. The CNN block was used to extract the features of lower-limb joint angles from the time series of the running standing phase and input these features into xLSTM for vertical-GRF prediction. When the seven datasets were input, CNN-xLSTM consistently showed the best performance among the four models used in this study (R^2^ = 0.909 ± 0.064, MAPE = 2.18 ± 0.09, rMSE = 0.061 ± 0.008). We also found in the prediction results that CNN-xLSTM has a better fitting effect on the double-peak characteristics of the vertical GRF during running in the rear-foot strike pattern [3,9] (Figure 4). In order to validate the robustness, we tested the four datasets with a relatively large contribution to the prediction results at five speeds (8 km/h, 10 km/h, 12 km/h, 14 km/h, and 16 km/h), and the results show that the prediction performance of CNN-xLSTM for the vertical ground reaction force was maintained at a relatively stable level at all five speeds, which indicates that the model was developed with good robustness (Figure 6, Table 3). In addition, CNN-xLSTM was compared with the three previous models mentioned in this paper that attempted to predict the vertical GRF, and the prediction performance of CNN-xLSTM was better than most models proposed by other studies that used acceleration as the input (Table 4). Although CNN-xLSTM has demonstrated good accuracy in prediction tasks, it should be acknowledged that the training and test datasets used by the models in the comparison are different from those in this study, and there may also be differences in the data collection methods. In previous studies, it may have been difficult to achieve good results in the prediction of the ground reaction force based on a single data point or feature. This study only used the angles of the lower-limb joints from the three planes and accurately predicted the vertical GRF during running. This result may depend on the analysis and judgment of the complex relationship between the joint angle data and the ground reaction force data in the time series by the ‘m’ and ‘s’ modules in xLSTM.

### 4.3. Prospects and Limitations

In this study, the vertical ground reaction force during running was predicted by using the angle of lower-limb joints in different motion planes by using deep learning methods. The results of this study provide a reference for future real-time motion detection and sport injury prediction. The joint angle can be used as a type of geometric data that can be collected and calculated by the image capture device in real time. At the same time, athletes or patients with sport injuries can adjust their stride length, cadence, and other sport modes in time according to the predicted results to achieve the purpose of improving sport performance and rehabilitation.

There are also limitations to this study. The study only collected data on lower-limb joint angles and vertical ground reaction forces during running from 12 healthy adult male runners, without considering the situation of female runners, and there is a lack of sample diversity that may affect the generality of the results. Additionally, when collecting data at five different speeds, due to equipment limitations, we did not strictly determine speed indicators, and there may be errors in the speed conversion results obtained from the photoelectric gates. Furthermore, the deep learning model developed in this study was not trained and tested using a public dataset, and results obtained using a public dataset may deviate from those in this study.

## 5. Conclusions

This study developed a CNN-xLSTM model and accurately predicted the vertical ground reaction force during the stance phase of running by inputting the joint angles of the lower limbs. The study also explored the impact of various joint angles on different planes on the prediction results. Additionally, we tested the predictive performance of the developed model across five distinct running speeds. The current findings may not only contribute to alternatives to the traditional mode of measuring the GRF with force plates in a laboratory but provide a reference for the setting of sport analysis equipment and the rehabilitation and training of athletes or sport injury patients.

## Figures and Tables

**Table 1:** Optimal parameter configuration of the four deep learning models used in this study.

Model	Batch Size	Hidden Size	Epochs	Stacked Layers	Module
CNN-xLSTM	128	64	25	1	‘m’, ’s’
CNN-sLSTM	128	64	25	1	‘s’
CNN-mLSTM	128	64	25	1	‘m’
CNN-LSTM	256	128	55	2	/

**Table 2:** The mean value and standard deviation of the R^2^, rMSE, and MAPE of 4 deep learning models trained by 7 datasets.

Models	Index	M1	M2	M3	M4	M5	M6	M7
CNN-xLSTM	R^2^	0.909 ± 0.064	0.814 ± 0.053	0.757 ± 0.092	0.709 ± 0.074	0.836 ± 0.042	0.861 ± 0.057	0.671 ± 0.051
	MAPE	2.18 ± 0.09	4.38 ± 0.21	9.95 ± 0.34	10.01 ± 0.41	3.17 ± 0.12	3.09 ± 0.18	12.82 ± 0.33
	rMSE	0.061 ± 0.008	0.089 ± 0.010	0.119 ± 0.014	0.132 ± 0.021	0.074 ± 0.007	0.070 ± 0.006	0.138 ± 0.017
CNN-sLSTM	R^2^	0.748 ± 0.056	0.702 ± 0.044	0.622 ± 0.069	0.656 ± 0.046	0.711 ± 0.055	0.729 ± 0.073	0.491 ± 0.067
	MAPE	3.71 ± 0.32	6.74 ± 0.59	17.06 ± 0.60	13.88 ± 0.77	6.27 ± 0.47	5.78 ± 0.58	15.06 ± 0.76
	rMSE	0.097 ± 0.011	0.114 ± 0.009	0.143 ± 0.014	0.161 ± 0.023	0.092 ± 0.007	0.094 ± 0.005	0.168 ± 0.028
CNN-mLSTM	R^2^	0.791 ± 0.027	0.713 ± 0.031	0.608 ± 0.035	0.627 ± 0.025	0.709 ± 0.031	0.721 ± 0.027	0.626 ± 0.031
	MAPE	5.56 ± 0.38	8.58 ± 1.04	19.49 ± 1.13	17.39 ± 0.93	9.19 ± 0.72	10.20 ± 0.97	16.38 ± 1.45
	rMSE	0.092 ± 0.012	0.112 ± 0.013	0.178 ± 0.032	0.209 ± 0.014	0.093 ± 0.013	0.096 ± 0.008	0.158 ± 0.024
CNN-LSTM	R^2^	0.742 ± 0.040	0.683 ± 0.032	0.619 ± 0.034	0.628 ± 0.041	0.728 ± 0.039	0.717 ± 0.041	0.653 ± 0.042
	MAPE	7.17 ± 0.45	8.12 ± 0.53	14.44 ± 1.05	15.07 ± 1.50	8.50 ± 0.80	8.89 ± 0.49	13.54 ± 0.98
	rMSE	0.104 ± 0.018	0.108 ± 0.006	0.135 ± 0.029	0.173 ± 0.037	0.112 ± 0.010	0.105 ± 0.008	0.172 ± 0.015

**Table 3:** The mean value and standard deviation of the R^2^, MAPE, and rMSE of CNN-xLSTM at 5 running speeds in the 4 cases that fitted the vertical-GRF curve better.

Inputs	Index	8 km/h	10 km/h	12 km/h	14 km/h	16 km/h
3 Joints and 3 Planes	R^2^	0.842 ± 0.047	0.854 ± 0.043	0.879 ± 0.068	0.861 ± 0.054	0.836 ± 0.032
	MAPE	2.45 ± 0.18	2.33 ± 0.09	2.19 ± 0.12	2.36 ± 0.11	2.71 ± 0.08
	rMSE	0.069 ± 0.012	0.067 ± 0.007	0.063 ± 0.010	0.065 ± 0.009	0.070 ± 0.013
Ankle and 3 Planes	R^2^	0.801 ± 0.037	0.804 ± 0.039	0.811 ± 0.036	0.807 ± 0.044	0.793 ± 0.043
	MAPE	4.69 ± 0.22	4.63 ± 0.26	4.45 ± 0.19	4.59 ± 0.18	4.78 ± 0.20
	rMSE	0.116 ± 0.018	0.109 ± 0.013	0.101 ± 0.011	0.105 ± 0.009	0.127 ± 0.021
3 Joints and Sagittal	R^2^	0.819 ± 0.62	0.821 ± 0.058	0.831 ± 0.055	0.829 ± 0.060	0.813 ± 0.052
	MAPE	3.73 ± 0.13	3.39 ± 0.15	3.24 ± 0.11	3.29 ± 0.12	3.87 ± 0.17
	rMSE	0.093 ± 0.019	0.089 ± 0.011	0.082 ± 0.012	0.085 ± 0.010	0.104 ± 0.014
3 Joints and Frontal	R^2^	0.811 ± 0.038	0.832 ± 0.050	0.857 ± 0.043	0.841 ± 0.037	0.803 ± 0.046
	MAPE	3.65 ± 0.21	3.27 ± 0.19	3.06 ± 0.13	3.12 ± 0.11	3.71 ± 0.16
	rMSE	0.091 ± 0.013	0.085 ± 0.017	0.079 ± 0.012	0.082 ± 0.012	0.093 ± 0.011

**Table 4:** The comparison of performance between CNN-xLSTM and the models mentioned in other studies that aim to predict the vertical GRF.

Studies	Models	Inputs	R^2^	MAPE (%)	rMSE
Oh et al. (2013) [13]	ANN	Centre of Mass and Acceleration of Segments and Joints	0.982	/	0.058 ± 0.010
Pogson et al. (2020) [25]	PCA-MLP-ANN	Trunk Acceleration	0.900	/	/
Alcantara et al. (2022) [29]	LSTM	Height, Mass, Speed, Slope, and Running Pattern	/	/	0.064 ± 0.015
Scheltinga et al. (2023) [24]	ensANN	Acceleration of Pelvis and Tibias	0.960	/	0.066 ± 0.001
Bogaert et al. (2024) [26]	Lasso	3-Dimensional Sacral Acceleration	0.870	3.29	0.106
Donahue et al. (2023) [30]	LSTM	3D accelerations and angular velocities	/	/	0.189
**This Study**	**CNN-xLSTM**	**Joints Angle of Ankle, Hip, and Knee**	**0.973**	**2.18 ± 0.09**	**0.061 ± 0.008**

## Data Availability

Data are contained within the article.

## Abbreviations

The following abbreviations are used in this manuscript:

GRF	Ground Reaction Force
IMU	Inertial Measurement Unit
CNN	Convolutional Neural Network
LSTM	Long Short-Term Memory
xLSTM	Extended Long Short-Term Memory
sLSTM	Scalar Long Short-Term Memory
mLSTM	Matrix Long Short-Term Memory
R^2^	Squared Correlation Coefficient
MAPE	Mean Absolute Percentage Error
rMSE	root Mean Squared Error
